# c-Myb Inhibits Myoblast Fusion

**DOI:** 10.1371/journal.pone.0076742

**Published:** 2013-10-21

**Authors:** Petr Kaspar, Kristina Ilencikova, Martina Zikova, Ondrej Horvath, Vladimir Cermak, Petr Bartunek, Hynek Strnad

**Affiliations:** Institute of Molecular Genetics, Academy of Sciences of the Czech Republic, Prague 4, Czech Republic; University of Minnesota, United States of America

## Abstract

Satellite cells represent a heterogeneous population of stem and progenitor cells responsible for muscle growth, repair and regeneration. We investigated whether c-Myb could play a role in satellite cell biology because our previous results using satellite cell-derived mouse myoblast cell line C2C12 showed that c-Myb was expressed in growing cells and downregulated during differentiation. We detected c-Myb expression in activated satellite cells of regenerating muscle. c-Myb was also discovered in activated satellite cells associated with isolated viable myofiber and in descendants of activated satellite cells, proliferating myoblasts. However, no c-Myb expression was detected in multinucleated myotubes originated from fusing myoblasts. The constitutive expression of c-Myb lacking the 3′ untranslated region (3′ UTR) strongly inhibited the ability of myoblasts to fuse. The inhibition was dependent on intact c-Myb transactivation domain as myoblasts expressing mutated c-Myb in transactivation domain were able to fuse. The absence of 3′ UTR of c-Myb was also important because the expression of c-Myb coding region with its 3′ UTR did not inhibit myoblast fusion. The same results were repeated in C2C12 cells as well. Moreover, it was documented that 3′ UTR of c-Myb was responsible for downregulation of c-Myb protein levels in differentiating C2C12 cells. DNA microarray analysis of C2C12 cells revealed that the expression of several muscle-specific genes was downregulated during differentiation of c-Myb-expressing cells, namely: ACTN2, MYH8, TNNC2, MYOG, CKM and LRRN1. A detailed qRT-PCR analysis of MYOG, TNNC2 and LRRN1 is presented. Our findings thus indicate that c-Myb is involved in regulating the differentiation program of myogenic progenitor cells as its expression blocks myoblast fusion.

## Introduction

Adult skeletal muscle is a terminally differentiated tissue, it nevertheless, retains an exceptional regenerative capacity that has been attributed to satellite cells, a heterogeneous population of stem and progenitor cells [1] localized between the basal lamina and sarcolemma of each muscle fiber. Following muscle injury, normally quiescent satellite cells, characterized by the expression of transcription factor Pax7, are activated and proliferate to create a pool of myoblasts which differentiate and fuse with the existing muscle fibers in order to repair the damaged segment or fuse together to create new muscle fiber. During proliferation and differentiation, satellite cells implement a skeletal myogenesis program that resembles embryonic myogenesis. Skeletal muscle development is controlled by coordinated up- and downregulation of myogenic regulatory factors (MyoD, Myf5, Myogenin and MRF4). Following activation, satellite cells leave their niche on the myofiber and move outside the basal lamina, re-enter cell cycle and express MyoD and Pax7. The descendants of activated satellite cells, myoblasts, proliferate and most of them downregulate Pax7 and differentiate expressing the differentiation markers MRF4 and Myogenin. In the process of injury repair, the quiescent satellite cell pool is also renewed.

c-Myb is a transcription factor with a DNA-binding domain, a central transactivation domain (TA) and a C-terminal negative regulatory domain [2]. c-Myb is required for modulation of progenitor cells in several tissues, including the adult brain [3], colonic crypts [4], the hematopoietic system [5] [6] and skin [7]. c-Myb plays a role in progenitor production, maintaining their proliferation, migration, or lineage commitment. Its expression generally declines as cells differentiate. c-Myb activity is tightly regulated at different levels, including downregulation by several miRNAs: miR-150 [8], miR-15a [9], miR-34a [10], miR-126 [11], miR-200b, miR-200c and miR-429 [12] binding to its 3′ UTR. As c-Myb is expressed in proliferating C2C12 cells and turned off in differentiating cells [13], we speculated that c-Myb could play a role in satellite cell biology.

We report here that c-Myb is expressed in activated satellite cells and proliferating myoblasts, and downregulated in myotubes. c-Myb constitutive expression strongly inhibits fusion of myoblasts. The inhibitory effect is dependent on intact transactivation domain of c-Myb and on the absence of 3′ UTR of c-Myb that contain several miRNAs binding sites. These results were verified using the satellite-cell derived myoblast cell line C2C12. In addition, using DNA microarray analysis of differentiating C2C12 cells several myogenic genes downregulated by c-Myb were identified. Accordingly, we suggest that c-Myb is suppressing the myogenic differentiation and its downregulation is a prerequisite for accomplishing the differentiation process.

## Materials and Methods

### Myofiber Isolation

Four-week old female BALB/c mice were sacrificed by cervical dislocation and myofibers were isolated from the extensor digitorum longus (EDL) muscle as described previously [14]. Briefly, an undamaged EDL muscle was dissected and digested with 0.2% collagenase type I (#C-0130, Sigma) in Dulbeccós modified Eaglés medium (DMEM) with 2% L-glutamine (Sigma) and 1% penicillin-streptomycin (Sigma) at 37°C in 5% CO2 for 60 min. Using a heat-polished Pasteur pipette, single fibers were removed and transferred to another plate with the same medium to take out debris before plating in 24-well plates (Nunc) coated with Matrigel (#356231, BD Biosciences). The isolated myofibers were intact and retained their associated satellite cells underneath the basal lamina. Generally, one fiber was placed per well.

### Cell Culture

Myofibers were incubated in a growth medium (DMEM supplemented with 2% L-glutamine, 10% horse serum, 0.5% chick embryo extract and 1% penicillin-streptomycin) or in an enriched growth medium (DMEM supplemented with 2% L-glutamine, 20% horse serum, 2.5% chick embryonic extract, 10 ng/ml basic fibroblast growth factor and 1% penicillin-streptomycin). For time-lapse analysis, DMEM without phenol red was used. C2C12 mouse myoblast cell line was obtained from ATCC and cultured as recommended. Cells were maintained in growth medium (GM) and differentiated in low serum medium (DM) as described in detail elsewhere [13].

### Muscle Injury

To induce regeneration of skeletal muscle, anesthetized 6-week old male BALB/c mice were injected with 100 µl of cardiotoxin (10 µM in PBS, #C9759, Sigma-Aldrich) into the left tibialis anterior (TA) muscle with a 30-gauge needle. The animals were handled in accordance with the Guide for the Care and Use of Laboratory Animals, approved by the Animal Care and Use Committee of the Academy of Sciences of the Czech Republic. Mice were kept under standard laboratory conditions with free access to food and water.

### Immunohistochemistry

Muscles were dissected from experimental animals, fixed in 4% paraformaldehyde-PBS overnight, embedded in paraffin and sectioned (7 µm). For immunohistochemical analyses, the sections were dewaxed, immersed in citrate buffer (10 mM, pH 6.0) and incubated for 25 min. at 98°C in a steam bath. After that, the slides were washed in PBS and incubated with 5% goat non-immune serum (Jackson ImmunoResearch) for 1 hour at RT to block nonspecific binding. For immunofluorescence double staining, the sections were incubated with polyclonal anti-Myb antibody (#AV38611, Sigma-Aldrich) at dilution 1∶200 at 4°C overnight and the next day, after three washings with PBS, incubated with anti-MyoD monoclonal antibody (#sc-32758, Santa Cruz Biotechnology) at dilution 1∶25 for 2 hours at RT. Secondary detection was performed using goat anti-rabbit biotin conjugate followed by Alexa Fluor 555 and goat anti-mouse Alexa 488 for 1 hour. The sections were mounted in ProLong Gold anti-fade reagent with DAPI (Life Technologies) to counterstain DNA.

### Retroviral Vectors

All DNA constructs were cloned into retroviral vector *pMSCV-IRES-eGFP*, a bicistronic vector expressing the gene of interest and eGFP from the same transcript (*pMSCV-gene of interest-IRES-eGFP*). The retroviral vector was kindly provided by Professor P. Zammit (King’s College London, London, UK). c-Myb expressing retrovirus (c-Myb-RET) was constructed by cloning a NcoI-NcoI fragment of mouse c-myb lacking the 3′ untranslated region, kindly provided by Dr. L. Wolff (NIH, Bethesda, USA), into the retroviral vector. To test whether both genes are expressed together in the same cell, C2C12 cells were infected with c-Myb-RET and immunostained for c-Myb and eGFP. Figure S1 shows co-expression of both genes in the same cell. The retrovirus expressing mutated c-Myb (c-Mybm-RET) was prepared using the same c-Myb cDNA except that it contained two point mutations encoding substitutions of leucine with alanine L302A and methionine with valine M303V in TA domain of c-Myb that were introduced with primer 5′-CTCGAGTTGCTCGCGGTGTCAACAGAGAACGA-3′ (new cloning XhoI site was also created by changing one nucleotide without changing amino acid composition). Mutations in TA domain of c-Myb abolish interaction with CBP/p300 and abrogate its transactivation and transforming ability [15]. In order to join the coding region of c-Myb with its 3′ UTR, part of the c-Myb coding region was amplified together with the whole untranslated region (NM_010848.3) from cDNA prepared from growing C2C12 cells using primers 5′-ACAGAGGACCAGATGACGGCCTCC-3′ (sense) and 5′-GCGTTAACAAGTATGTAAAATAAGAGGATAGC-3′ (antisense) (HpaI site was introduced). In several cloning steps, the c-Myb coding region with 3′ UTR was introduced into retroviral vector (c-Myb-3′U-RET). To generate miR-150 expressing retrovirus (miR-150-RET), the genomic PCR product (166 bp), containing the entire pri-miR-150 sequence, was obtained using 5′-AGCAGTGCTTTCCGCAGCATC-3′ (sense) 5′-GTCCCTTGGCTGGAGGGAGAA-3′ (antisense) primers and cloned into the retroviral vector. All PCR-derived sequences were confirmed by sequencing. As a control, we used empty retrovirus *pMSCV-IRES-eGFP* (control-RET).

### Western Blotting

Western blotting was performed as described previously [13]. Protein concentration was determined by BCA TM Protein Assay Kit (Thermo Scientific, Pierce). Twenty-five micrograms of the cell extract was analyzed by monoclonal anti-Myb antibody (#05-175, clone1-1, Millipore) at dilution 1∶500 according to the manufacturers instructions or polyclonal anti-Myb antibody (#AV38611, Sigma-Aldrich) at dilution 1∶1000 and 1 µg of extracts was analyzed by monoclonal anti-GAPDH antibody (#GTX30666, GeneTex) at dilution 1∶2000.

### Cell Sorting

Cells were detached from culture dishes with trypsin-ethylenediaminetetraacetic acid (EDTA). For cell sorting, cells were transferred to the growth medium and eGFP-expressing cells were separated with BD Influx TM cell porter (BD Biosciences, NJ, USA).

### Immunofluorescence Staining

C2C12 cells were immunostained as described previously [13]. Immunofluorescent staining for c-Myb visualization was performed using monoclonal anti-Myb antibody (#05-175, clone 1-1, Millipore) as described [16]. The following primary antibodies were used: polyclonal anti-MyoD antibody (#sc-760, Santa Cruz Biotechnology), monoclonal anti-Myosin heavy chain (MHC) antibody (MF 20, Developmental Studies Hybridoma Bank) and polyclonal anti-GFP antibody (#ab 290, Abcam). Cultured myofibers were fixed in 4% paraformaldehyde/PBS for 15 min, permeabilized in 0.5% Triton X-100/PBS for 10 min, blocked with 2% BSA (#15260, Gibco) for 1 hour and incubated overnight with the same primary antibodies as described for C2C12 cells. Fluorochrome-conjugated antibodies (Abcam) were used to visualize the primary antibodies. Fluorescence images were captured using a DMIRB microscope, DFC480 camera and IM500 software (Leova Microsystems, Wetzlar, Germany) and processed using Adobe software (Adobe Systems, San Jose, CA, USA).

### qRT-PCR

qRT-PCR reactions were performed in triplicates using FastStart SYBR Green Master (Roche Applied Science, IN, USA) and analyzed by using the Light Cycler® 480 Instrument II (Roche Applied Science). GAPDH was used to normalize the RNA content of samples and the comparative ΔΔC(t) method was employed to calculate relative expression levels. qRT-PCR primers are listed in Table S1. To quantify miR-150, qRT-PCR was performed in triplicates using TaqMan MicroRNA assays (Applied Biosystems) assay ID: 000473 according to manufacturers instructions. Relative expression of miR-150 was calculated using ΔΔC(t) method (normalized to corresponding U6 snRNA values, assay ID: 001973).

### Microarray Analysis

The microarray analyses were performed with RNA isolated from C2C12 cells expressing c-Myb, miR-150 and control C2C12 (infected with empty retrovirus) at three time points: cells cultured in GM were harvested before transferring to differentiation medium (D0), after cultivation in the differentiation medium for 24 hours (D24) and 72 hours (D72). The experiments were carried out in triplicate (each experiment started with retroviral infection of C2C12 cells, then eGFP sorting was performed, followed by differentiation of infected C2C12 cells). Total RNA was isolated using TRI Reagent (#TR118, Molecular Research Center, OH, USA) and checked for integrity. 150 ng of total RNA was amplified with Illumina TotalPrep RNA Amplification Kit (Ambion, TX, USA) and 750 ng of amplified RNA was hybridized on an Illumina MouseRef-8 v2 Expression BeadChip (Illumina, USA). All steps were done according to manufacturers instructions. The raw data was analyzed and processed using the bead array package of the Bioconductor within the R environment (R Development Core Team, 2007). The data was deposited to the ArrayExpress database under the accession number E-MTAB-1376 X.

### Time-lapse Microscopy

Myofiber cultures were placed on the motorized stage of a Leica DMI 6000B microscope, enclosed by a BL109 incubator (PeCon GmbH) and cultured at 37°C, 5% CO2. Time-lapse microscopy using a 10×objective lens was taken at the rate of one frame every 3 minutes for up to 72 hours using a Leica DFC360 FX camera equipped with 488 nm laser. Time lapse image data were corrected for bleaching, lamp jitter, field flatness and 5×5 low pass filtered (Huygens SW, SVI, Netherlands and MetaMorph SW, Molecular Devices, USA) to improve the object detection. Cell motility was subsequently analyzed using a Huygens SW object tracker.

### Migration Assays

Migration of C2C12 cells was monitored by scratch wound healing assay and time-lapse microscopy or by transwell migration assay. Transwell migration assay was performed using 8-µm pore size inserts (#351152, BD Falcon). C2C12 cells were plated at a density of 1×10^5^ cells/insert in GM in the upper chambers and allowed to migrate for 6 hours, the bottom chambers contained the same growth medium. Inserts were then removed from a 24-well plate and the cells on the upper side of the insert were removed with a cotton swab. The cells on the lower side of the insert were fixed with 4% paraformaldehyde, washed, and stained by the DAPI and counted.

### Determination of Cell Growth Kinetics

C2C12 cell numbers were determined by counting cells with cell counter CASY® (Shärfe Systems GmbH) or the numbers of viable cells were determined by CellTiter -Blue® Cell Viability Assay (Promega).

## Results

### c-Myb is Expressed during Skeletal Muscle Development and Regeneration

We were interested in finding whether c-Myb plays a role in satellite cell biology. We therefore employed an experimental model that is based on the isolation of viable muscle fibers carrying satellite cells in their niche on the fiber which provides signals maintaining their stem cell specification. During ex vivo cultivation of isolated myofiber on Matrigel, which contains a mixture of basement membrane components and growth factors, satellite cells are activated, migrate from the fiber, the resulting myoblasts proliferate and fuse together to form multinucleated myotubes. We performed cultivation of isolated myofibers in high serum medium (10% horse serum) resulting in quick activation of associated satellite cells and an initiation of process mimicking in vivo regeneration [17]. As isolated myofibers tend to contract after isolation, we cultured isolated myofibers for 24 hours on Matrigel (about 50% of myofibers during 24 hours of cultivation collapsed) and the remaining myofibers were firmly attached to Matrigel and could be cultivated for a period up to one week. Movie S1 documented all of the steps of muscle differentiation, i.e. satellite cell activation and migration from the myofiber, proliferation and fusion of myoblasts. The number of emigrating satellite cell was low and we usually detected about three to five cells. However, cultivation of isolated fibers in enriched growth media increased the number of emigrating activated satellite cells as well as the proliferating period before myoblast fusion. We also noted that proliferating myoblasts did not escape from the myofiber vicinity, suggesting they were subjected to signals emanating for the trauma-suffering myofiber. That is why the newly formed myotubes sometimes had a tendency to fuse with the parental myofiber as they may have been responding to myofiber signaling. To test the viability of myofiber cultures, we performed immunofluorescence analysis to examine whether myoblasts were presented in myofiber cultures cultivated for a longer time. Figure S2 documented that myoblasts (MyoD-positive) proliferated and fused to myotubes. In myofiber cultures cultivated for five days, myotubes were rare but they accumulated gradually as after 7 days of culturing there were many myotubes visible. The myotubes first appeared in the vicinity of myfiber because the highest myoblasts concentration was there while the culture continued to expand at the edges and myoblasts proliferated robustly even after 7 days in culture.

**Figure 1:**
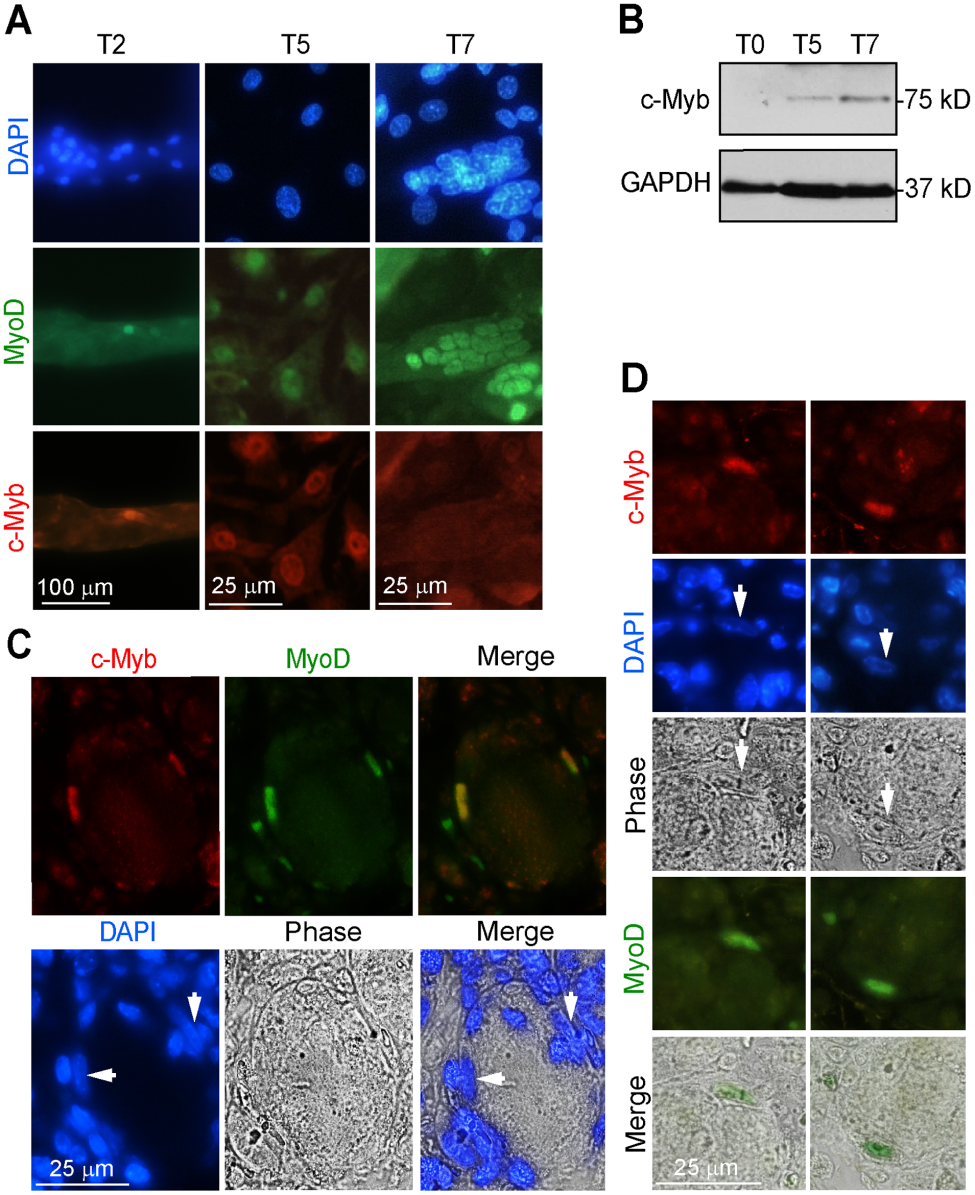
Endogenous expression of c-Myb during the skeletal muscle development and regeneration. Myofibers from the EDL were isolated and plated on Matrigel. This procedure enables activated satellite cells to emigrate from the myofiber proliferate and fuse into multi-nucleated myotubes. After 1–2 days of culturing in enriched growth medium, the activated satellite begin to emigrate from myofiber and the arisen proliferating myoblasts start to differentiate after 5 days. (A) Myofibers were cultured from two days to seven days and analyzed for expression of c-Myb and MyoD by immunofluorescence using specific antibodies. After two days (T2), fiber-associated satellite cells were immunostained; after 5 days (T5) proliferating myoblasts and after 7 days (T7) myotubes were immunostained. Nuclei were counterstained by DAPI. We note that endogenous c-Myb is expressed in activated satellite cells, proliferating myoblasts but not in myotubes. (B) Total cell extracts from freshly isolated myofibers (T0) and from myofibers cultivated on matrigel for five days, (T5) and seven days (T7) were analyzed by Western blotting for c-Myb expression. GAPDH served as an internal control. c-Myb was detected on Western blots of extracts of cultivated myofibers from which the satellite cell migrated and resulting myoblasts proliferated extensively. The representative results of three independent experiments are shown. (C, D) Immunohistochemical detection of c-Myb and MyoD on paraffin-embedded sections of TA muscle regenerated for 5 days after cardiotoxin injection. (C) MyoD expression marks the activated satellite cell located beneath the basal lamina of a newly formed myofiber. Immunostaining demonstrated colocalization of cMyb and MyoD. (D) On selected muscle cross sections, membranes structures surrounding MyoD and c-Myb positive satellite cell were preserved.

Next, we examined whether satellite cells expressed c-Myb during their lifetime. For this reason, we performed an immunofluorescence analysis for c-Myb expression. We identified c-Myb in activated satellite cells associated with myofiber and in proliferating myoblasts, however, the myotubes were negative for c-Myb (Figure 1A). The expression of c-Myb in myoblasts proliferating for 5 days (T5) and 7 days (T7) was confirmed by Western blotting (Figure 1B).

Next, we performed an immunohistochemical analysis (IHC) of paraffin-embedded muscle tissues induced to regenerate by cardiotoxin injection for c-Myb. The cardiotoxin injection caused degeneration of muscle tissue which was followed by an active muscle regeneration period during which satellite cells divided and repaired the damaged muscle. Regenerated muscle fibers appeared 5–7 days after cardiotoxin injury and were recognized by centrally localized nuclei [18]. In our muscle injection experiments the regeneration process was executed at the same speed (Figure S3). We therefore performed IHC on the histological section of TA muscle 5 days after the cardiotoxin-induced injury. The result shown in Figure 1C demonstrate MyoD expression in mononuclear cells located beneath the basal lamina of newly formed myofiber that is consistent with expression in activated satellite cells. In addition, the co-localization of MyoD and c-Myb expression was shown indicating that activated satellite cells express c-Myb. Satellite cells are mononuclear cells with low cytoplasmic content localized between the basal lamina and plasmolemma of each muscle fiber. In Figure 1D, we present the IHC of histological sections in which membranes surrounding satellite cells were visible. It was shown that the satellite cells, localized inside membrane structure, were immunostained for MyoD and c-Myb.

### c-Myb Prevents Fusion of Myoblasts

Our data indicate that c-Myb is expressed in myogenic progenitor cells and downregulated in forming myotubes. To investigate the potential role of c-Myb in myogenic progenitor cells, we decided to modulate c-Myb expression in progenitor cells. To upregulate c-Myb expression, we infected cultured myofibers with retroviral vector *pMSCV-cMyb-IRES-eGFP* (c-Myb-RET) expressing c-Myb as a bicistronic transcript with eGFP. As it is documented that c-Myb is subjected to downregulation via several miRNAs targeting its 3′ untranslated region, we expressed only the coding sequence of c-Myb to avoid possible interactions with miRNAs. To downregulate endogenous c-Myb levels, we used miR-150-expressing retrovirus *pMSCV-miR-150-IRES-eGFP* (miR-150-RET) as miR-150 efficiently inhibits c-Myb expression. Moreover, miR-150 is expressed at low levels in C2C12 cells [19] and is slightly upregulated in C2C12 cells differentiating for 3 days [20] (See supplementary data). In order to confirm the published data, we analyzed miR-150 expression in growing and differentiating C2C12 cells and found that it was upregulated on the first day of differentiation (Figure S4). Considering c-Myb expression having been silenced at the same time, we speculated that miR-150 could play a role in c-Myb downregulation during skeletal muscle development. Empty retrovirus *pMSCV-IRES-eGFP* (control-RET) was used as a control.

We infected cultured myofibers with retroviruses c-Myb-RET, miR-150-RET and control-RET at a time when about 10 myoblasts were identified around the fiber (it usually takes two or three days of culturing the myofiber in enriched growth medium). Satellite cells still associated with myofiber and proliferating myoblasts were exposed to the retrovirus for 24 hours and then, after washing out the virus, were cultivated for another day to allow infected cells to multiply. Finally, the selected region containing myofiber surrounded by the largest number of infected cells was analyzed by time-lapse microscopy. Cells co-expressing retroviruses were easily monitored, as eGFP-positive cells visible in fluorescence images that were taken together with phase contrast images every 3 minutes. Time-lapse microscopy was performed at least for two days. The entire time-lapse movie is provided as the merged images of phase contrast and eGFP fluorescence images. In Movie S2, cultured myofibers were infected with c-Myb-RET and in Movie S3 with control-RET.

**Figure 2:**
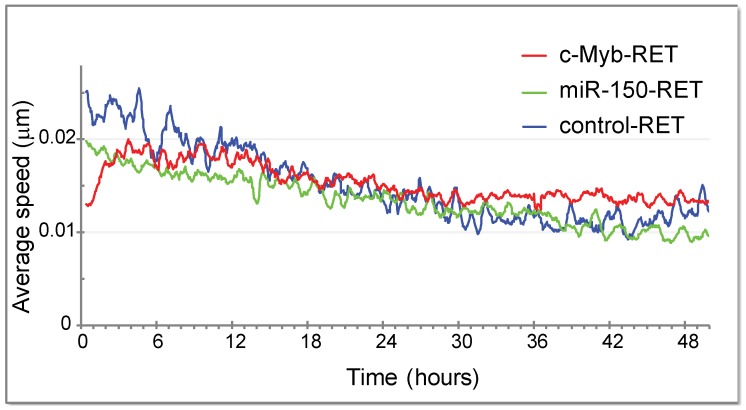
Average speed of myoblasts infected with retroviruses cultured on Matrigel. c-Myb-RET, miR-150-RET and control-RET retroviruses were used to infect myoblasts arisen from cultured myofibers. eGFP positive myoblasts were monitored by time lapse microscopy for two days and analyzed for migration using a Hugens SW object tracker. The average speed of myoblast movement between individual time points is shown.

**Figure 3:**
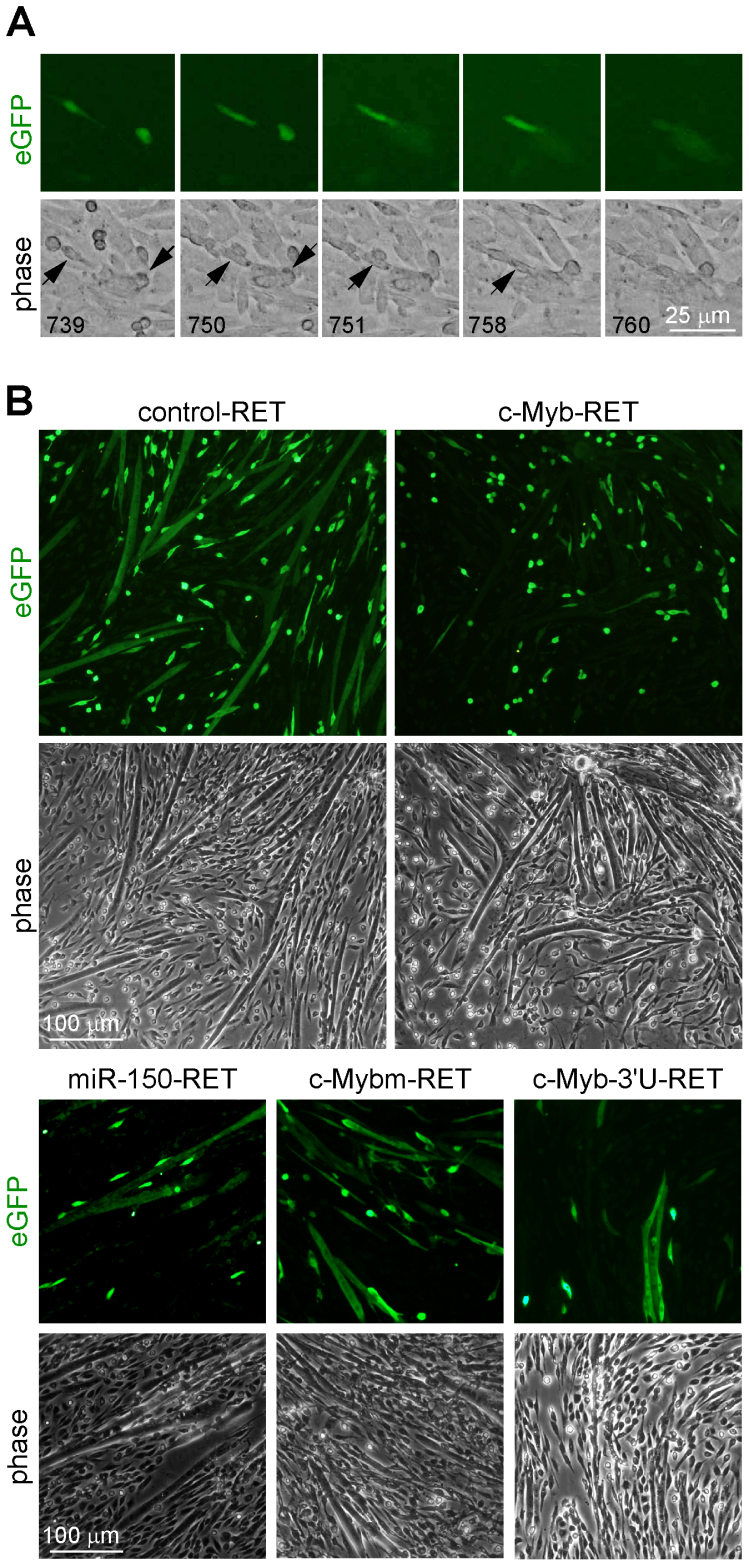
c-Myb expressing myoblasts do not form myotubes. (A) Single frames illustrating the fusion of two eGFP+ myoblasts (black arrow) with arising myotube. Isolated myofibers were plated, and after 2 days of culturing in enriched growth medium, infected with control-RET retrovirus. After 24 hours, the virus was removed and time-lapse microscopy was performed. Phase contrast images (the frame number is shown) and the corresponding fluorescence images (eGFP) were taken every 3 minutes for a period of 3 days. (B) Alternatively, myoblasts were exposed to control-RET, c-Myb-RET, mir-150-RET, c-Mybm-RET and c-Myb-3′U-RET retroviruses, cultivated for 3 days after removing retroviruses and immunostained for eGFP. We note that the only infection with c-Myb-RET results in eGFP positive myoblasts that do not contribute to myotube formation as myotubes are devoid of eGFP. Representative results of three independent experiments are shown.

The images were analyzed for migration of eGFP positive cells and Figure 2 graph shows average speed of myoblast movement between individual time points. We found that the average speed was about the same for all constructs, slightly decreasing with time, as cell density was increasing. The only significant difference was seen in the first 6 hours of the experiment, but the reason was likely the unstable conditions at the beginning of experiment (temperature, CO_2_ concentration); with time, as conditions stabilized, the differences in speed disappeared. As c-Myb also stimulates proliferation in various cell types, we expected that satellite cell-derived myoblasts expressing c-Myb would proliferate faster as well. Therefore, we analyzed all three movies by tracing a single cell and counting the proliferation (cell-cycling) time between cell divisions. We found that there were negligible differences in the time between cell divisions for positive, infected cells (eGFP^+^) and negative, non-infected cells (eGFP^−^) no matter which retroviral construct was used. c-Myb-RET infected eGFP^+^ cells were 0.95 times faster than c-Myb-RET non-infected cells (eGFP^−^), cells whose proliferation rate was set to 1. miR-150-RET infected eGFP^+^ cells were equal with non-infected eGFP^−^ cells as well as control-RET. Our data thus indicates that c-Myb in myogenic progenitor cells influences neither migration nor proliferation, the processes c-Myb is involved in other cells types. However, a detailed inspection of movies revealed that c-Myb-expressing myoblasts do not fuse. In contrast, the infection with control-RET and miR-150-RET generated eGFP^+^ myoblasts that can fuse together, the fusion was often observed and one example of cell fusion is documented on Figure 3A showing single frames illustrating the fusion of two eGFP^+^ myoblasts (black arrow) with arising myotube. The low fluorescence signal is also detectable in several myotubes on Movie S3 where cultures were infected with control-RET. To verify these findings, we performed the same experiment but after infection we cultivated myofibers for another three days and immunostained myotubes for eGFP. We included in the experiment two other retroviral constructs, c-Mybm-RET with mutated c-Myb in TA domain and c-Myb-3′U-RET, where coding sequence of c-Myb was followed by its 3′ untranslated region with binding sites for miRNAs. Figure 3B shows that infection with all retroviral vectors, except for c-Myb-RET, resulted in forming of eGFP^+^ myotubes. We conclude that constitutive expression of c-Myb lacking its 3′ UTR blocks myoblast fusion. The inhibition is dependent on the intact transactivation domain of c-Myb and on the absence of 3′ UTR of c-Myb that is likely to induce c-Myb degradation.

### c-Myb Prevents Fusion of C2C12 Cells

C2C12 is a mouse myoblast cell line frequently used as an experimental model for studies of myogenic differentiation. These cells, which are induced to differentiate into myotubes in low serum medium, represent a suitable experimental model to verify the effect of c-Myb on myogenic progenitor cells. We infected C2C12 cells with the same retroviral constructs that were used in experiments with cultured myofibers. After exposure to the retroviruses for 24 hours, the cells were sorted for eGFP, positive cells were collected, cultured in GM and differentiated in DM.

**Figure 4:**
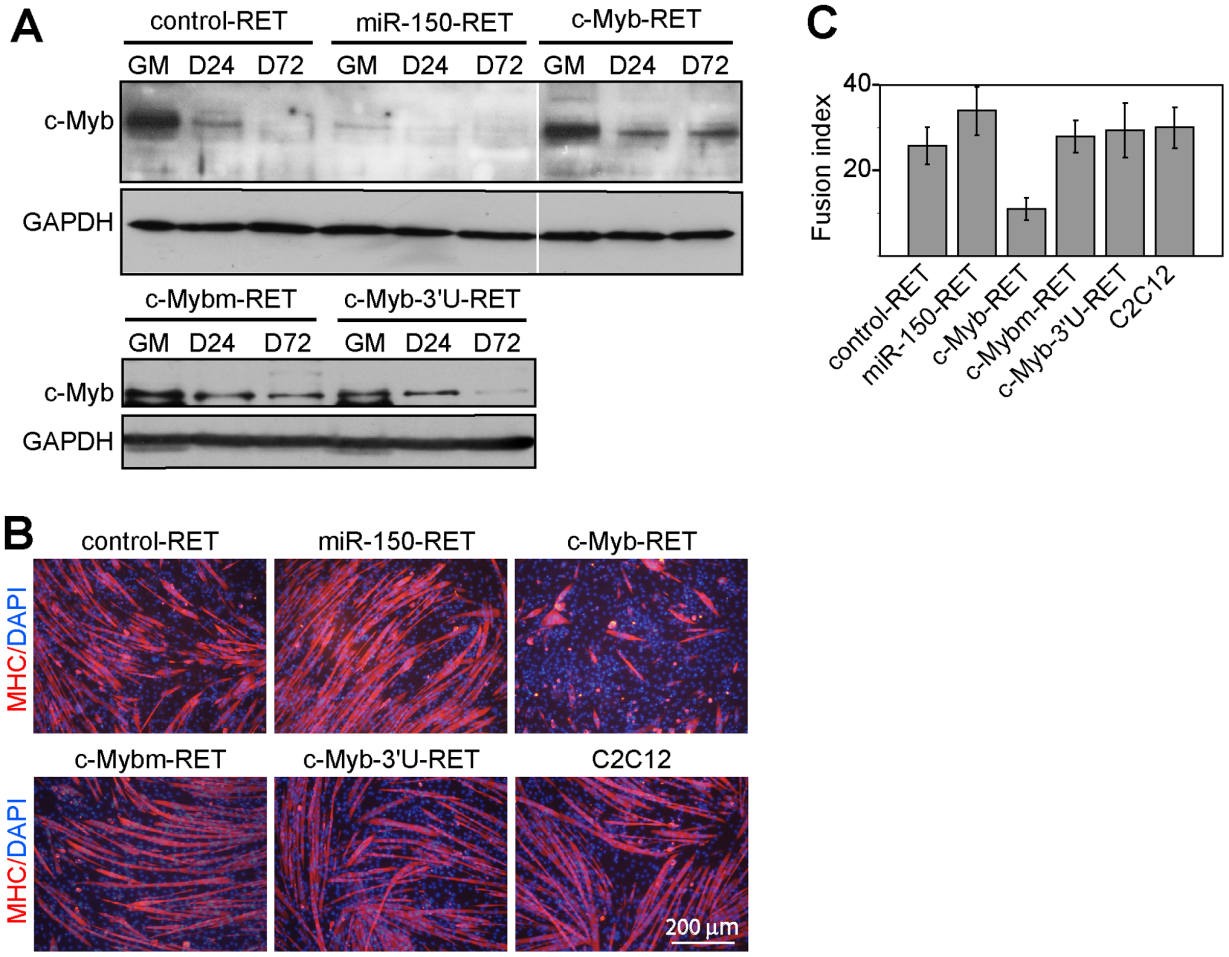
c-Myb reduces the ability of differentiating C2C12 cells to fuse into myotubes. C2C12 cells were infected with control-RET, c-Myb-RET, miR-150-RET, c-Mybm-RET and c-Myb-3′U-RET retroviruses, sorted by FACS 24 hours later to select for eGFP-positive cells, and induced to differentiate by incubation in DM. (A) Western blotting showing expression of c-Myb in lysates from retroviruses-infected C2C12 cells cultured in growth condition (GM) and differentiated in DM for 24 hours (D24) and 72 hours (D72) before harvesting. GAPDH served as an internal control. (B) Representative image of immunofluorescence staining for myosin heavy chain (MHC) after 3 days cultivation in DM. Nuclei were counterstained by DAPI. (C) Quantitative analysis of myotube formation. The graph shows the fusion index which is defined as the percentage of nuclei belonging to MHC-positive cells with two or more nuclei. 1000 nuclei/field were counted from 7 randomly selected fields. The values reported in the graph are the means±standard deviations (S.D.). Statistical analyses of the data were performed using the Student’s t-test. A P value of less than 0.05 was considered statistically significant for all analyses. We note that c-Myb expression during differentiation reduces the ability of cells to fuse into multinucleated myotubes. Representative results of three independent experiments are shown.

We analyzed c-Myb expression on both transcriptional (not shown) and translational levels. While analysis of c-Myb transcription levels resulted in expected findings (high constitutive transcription of c-Myb and its variants from integrated retroviruses, downregulation of endogenous c-Myb mRNA during cultivation of cells in DM, reducing of c-Myb mRNA levels in miR-150-RET infected cells), the analysis of c-Myb protein levels by Western blotting (Figure 4A) resulted in one unexpected finding: c-Myb-RET (and c-Mybm-RET as well) infection did not increase c-Myb protein levels in GM. However, in DM, we identified continuous expression as we expected. In cells infected with the miR-150-RET, only the traces of c-Myb were detected in GM indicating that miR-150 was suppressing c-Myb expression on both transcriptional and translational levels. In c-Myb-3′U-RET infected cells, c-Myb was downregulated in DM at a lower speed than in control cells infected with control-RET. At D72, however, no expression was detected indicating that 3′ UTR was responsible for c-Myb degradation.

To assess the inhibition potential of c-Myb on fusion of individual cells into myotubes, we estimated the fusion index, which is the percentage of all myonuclei presented in myotubes, for all retroviral constructs. We differentiated infected eGFP^+^ C2C12 cells in DM for three days and, after immunostaining for MHC to visualize myotubes, the formation of myotubes was quantified by calculating the fusion index. Figure 4B+C illustrates that c-Myb-RET infection caused a profound reduction of myotube formation. The inhibitory effect of c-Myb on myoblast fusion was thus confirmed in C2C12 cells as well.

Next, we examined whether migration of C2C12 cells with low expression of c-Myb (infection with miR-150-RET) was affected, compared to cells with physiological expression of c-Myb in GM (C2C12 cells) or cells infected with c-Myb-RET, but we found no difference in mobility. The growth kinetics were also equal as well as cell-cycle progression measured by flow cytometry [13] (also measured in DM). These results are in agreement with data obtained using cultured myofibers.

### Microarray Analysis

As we were able to downregulate the extent of myotube formation by increasing c-Myb levels, we were interested in finding whether there are muscle-specific genes responding to changes in c-Myb expression. We performed a microarray analysis of infected C2C12 cells at three time points to monitor the course of differentiation: in GM, after 24 hours in DM (D24) and after 72 hours in DM (D72). We searched only for genes that are differently expressed in c-Myb-RET cells in comparison with miR-150-RET cells (the difference in gene expression 1.7 times or more in at least one time point). All genes that fulfilled the above criteria were downregulated in c-Myb expressing cells and at the same time upregulated in cells not expressing c-Myb. There are 6 genes on the list that are muscle-specific, namely: ACTN2, MYH8, TNNC2, MYOG, CKM and LRRN1.

**Figure 5:**
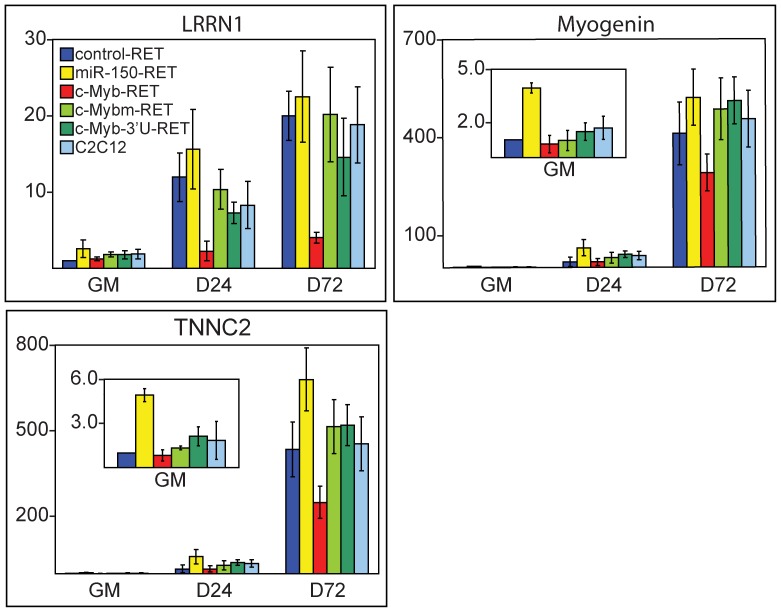
Genes downregulated by c-myb during muscle differentiation of C2C12 cells. qRT-PCR analysis of genes identified by microarray analysis as the most inhibited genes by c-Myb. Myogenin, TNNC2 and LRRN1 relative expression in cells infected with various retroviral constructs was determined at three time points, in GM and after differentiation in DM for 24 (D24) and 72 hours (D72). Bars represent average values of relative expression with respect to the average expression level of the mRNA in normal growth conditions (GM) in cells infected with control-RET. GAPDH was used to normalize the RNA content of the samples. The averages were calculated from two (GM) or three (D24 and D72) biological replicates and each measured as three technical replicates. Error bars show combined standard deviations (technical plus biological).

Next, we performed a qRT-PCR analysis of identified genes in cells infected with all retroviral constructs. qRT-PCR analysis confirmed the results of the microarray data. In Figure 5, the representative analysis of the highly inhibited genes MYOG, TNNC2 and LRRN1 is shown. MYOG encodes Myogenin, an important myogenic regulatory factor promoting conversion of myoblasts to myotubes, TNNC2 encodes the troponin C2 protein involved in muscle contraction and LRRN1 encodes a transmembrane protein expressed during embryonal myogenesis. All inspected genes were downregulated by constitutive c-Myb expression in DM that was attained only with c-Myb-RET construct and the inhibition was again dependent on the c-Myb intact TA domain. On a side note, the absence of c-Myb in GM (miR-150-RET) resulted in upregulation of the same set of genes as was inhibited by the c-Myb excess in DM, but there is a possibility that miR-150 could affect the expression profile of the inspected genes in GM as well. Our analysis thus identified several muscle-specific genes that were inhibited by c-Myb during myogenic differentiation.

## Discussion

To analyze the role of c-Myb in satellite cells, we used cultured myofibers and C2C12 cells. During the cultivation of myofibers, the associated satellite cells were activated and completed the myogenic differentiation program. We identified c-Myb expression in activated satellite cells in their niche on the myofiber and in proliferating myoblasts, but not in multinucleated myotubes. The activated satellite cells in regenerating muscle tissue were also c-Myb positive. Similarly, in C2C12 cells, c-Myb was expressed in growing cells and turned off in differentiated myotubes.

We upregulated c-Myb by using retroviral construct and the high constitutive transcription was documented, but protein levels were low, almost equal to endogenous levels in control cells in GM. Western blotting revealed that at all the time points in c-Myb-expressing cell extracts, many extra bands were detected of lower molecular weight than was the expected for the size for c-Myb. These extra bands were not detected in other samples not overexpressing c-Myb such as, for example, in control-RET infected cells (not shown in Figure 4A). We think that these bands represent degradation products of c-Myb as cells degrade the excess of c-Myb. However, the reduced expression of c-Myb persisted c-Myb-RET infected cells during differentiation in DM while the endogenous c-Myb had already been turned off. We presume that a similar degradation of excess of c-Myb could also happen in proliferating myoblasts in cultured myofibers (immunostaining showed that eGFP^+^ myoblasts did not express high levels of c-Myb). As the infected cells do not overexpress c-Myb in GM, we cannot see any effect c-Myb on proliferation and migration of myoblasts or C2C12 cells in GM.

By expressing miR-150, we successfully reduced c-Myb levels. The fact that we created cells devoid of c-Myb that are indistinguishable in GM from parental C2C12 cells is rather surprising. We discovered that proliferation and migration was not influenced by low c-Myb levels again, indicating c-Myb is not involved, but we have to take into consideration the fact that cells also overexpress miR-150 and we cannot exclude the possibility that it could influence the proliferation and migration processes in an opposite way than the lack of c-Myb.

While in GM conditions, the modulation of c-Myb expression did not lead to an apparent effect while during differentiation, its detrimental effect was clear. Our c-Myb construct ensured low but continuous expression of c-Myb during differentiation. As cells began to fuse they were becoming extremely sensitive to c-Myb and did not fuse if c-Myb was presented even in reduced levels as in our experimental setup. Constitutive expression of c-Myb led in both experimental systems to strong inhibition of cell fusion. The quantification of the inhibitory effect of c-Myb on myotube formation is impossible by using cultured myofibers as we cannot calculate the fusion index for eGFP^+^ cells and immunofluorescence staining for eGFP only roughly reflects the number of fused eGFP^+^ cell in forming myotubes. Nevertheless, in combination with immunostaining for eGFP and the visual inspection of movies it seems that the inhibition was almost absolute as no myoblast with deregulated expression of c-Myb was able to fuse. In the C2C12 cells, where the extent of myotube formation can be easily calculated on the basis of fusion index, it was shown that the inhibition was only partial leading to fusion index 11% in C2C12 cells expressing exogenous c-Myb compared to 28% in control cells. These results indicate that c-Myb negatively regulate myotube formation. In order to find some clues on how c-Myb inhibits myoblast fusion, we employed c-Myb mutant with mutations in TA domain that disrupted interaction with CBP/p300. We revealed that mutated c-Myb was unable to suppress the fusion process.

Next, we performed a DNA-microarray analysis. We searched for genes that are expressed differently (≥1.7fold change) in cells overexpressing c-Myb (c-Myb-RET infected cells) in comparison with cells devoid of c-Myb (miR-150-RET infected cells). 20 genes fulfilled this criterion, 6 of which were muscle-specific. All identified genes were downregulated in c-Myb expressing cells. We did not identify any gene that was activated in c-Myb expressing cells. Some of the identified muscle-specific genes, namely: TNNC2, MYOG, and LRRN1, were also significantly upregulated in cells devoid of c-Myb in GM. These results indicate that the expression of the indicated genes is modulated by c-Myb, suggesting that c-Myb inhibits their transcription. However, there is a possibility that miR-150 could also affect the expression of the identified genes in GM. We also showed that the inhibitory effect of c-Myb on skeletal myogenesis is eradicated when the TA domain contains two point mutations disrupting the interaction with CBP/p300. One possible explanation of our findings is the speculation that c-Myb has a role as a transcriptional activator during skeletal myogenesis. c-Myb may associate with several co-activators and activate c-Myb-responsive gene(s) that consequently repress transcription of muscle-specific genes; CBP/p300 may play a prominent role in this process, and c-Myb mutant abrogating CBP/p300 binding is therefore defective in activating c-Myb-responsive genes. However, the analysis of our microarray data, did not reveal c-Myb-responsive genes that could play such a role. Alternatively, c-Myb could also be associated with co-repressors. It was documented that several co-repressors can bind c-Myb, namely: TIF1β, mSin3A, c-Ski, and N-CoR [21]. Those co-repressors interact with each other and form macromolecular complexes with class I and II histone deacetylases (HDAC) that are recruited to c-Myb. It is suggested that the association with co-repressors and histone deacetylases suppress the potential oncogenic properties of c-Myb. We can speculate that c-Myb associated with co-repressors and HDAC could play a transcriptional co-repressor role in proliferating myogenic progenitor cells. In support of the co-repressor role of c-Myb is the discovery that Myb-binding protein 1a (Mybbp 1a) suppresses myogenesis [22]. Mybbp 1a was originally identified as a protein able to interact with a leucine zipper-like motif in the negative regulatory domain of c-Myb. In proliferating myoblasts it inhibits expression of muscle-specific genes by associating with HDACI/II and stabilizing the co-repressor complex to the promoters of muscle-specific genes inducing less permissive chromatin structure and gene silencing. At the onset of differentiation, expression of Mybbp 1a is downregulated, leading to destabilization of co-repressor complexes and replacement with co-activators and subsequent gene activation. There is a possibility that c-Myb associated with co-repressors and HDACI/II becomes a component of transcriptional co-repressor complex by interacting with Mybbp 1a via leucine zipper structure. This would mean that the same genes were inhibited in C2C12 cells expressing c-Myb compared to Mybbp1a expressing cells. Our results indicate that expression Myogenin, TNNC2 and LRRN1 is inhibited by c-Myb, as it is upregulated in c-Myb-knockdown cells and suppressed in cells overexpressing c-Myb. The same expression pattern was observed for Myogenin in cells with modulated expression of Mybbp1a [22] (upregulation in Mybbp1a-knockdown and downregulation in cells overexpressing Mybbp1a) and partly for TNNC2, the expression of which was upregulated in Mybbp1a-knockdown cells [22] (see Supplementary data). Moreover, we found by inspecting our microarray data that several muscle-specific genes identified as Mybbp 1a targets are also potential targets for c-Myb repression as they are upregulated in c-Myb-knockdown and downregulated in cells overexpressing c-Myb, but the microarray data were not verified by qRT-PCR. Finally, both Mybbp 1a- and c-Myb-knockdown cells differentiate at a higher speed than control cells and the two genes are subjected to post-transcriptional downregulation by miRNAs and silenced during myogenic differentiation. Thus, it seems that c-Myb and Mybbp 1a could cooperate in regulating the onset of myogenic differentiation. However, our experiments also involved c-Myb mutant in the TA domain which did not exhibit inhibition of muscle-specific genes. If we consider c-Myb as a component of transcriptional co-repressor complex, this would mean that c-Myb with point mutations in the TA domain that abrogated binding to CBP/p300 did not interact with Mybbp 1a or the forming of c-Myb complex with co-repressors and HDAC was prevented. One possible explanation is that mutations in the TA domain change the conformation of c-Myb in such a way that the interaction between c-Myb and Mybbp 1a or c-Myb and co-repressors and HDAC was abolished. It seems that especially the binding of c-Ski to DNA binding domain of c-Myb could be affected as its binding is sensitive to changes in negative regulatory domain. It is suggested that deletion or mutation of the negative regulatory domain change the conformation of c-Myb protein resulting in the TA domain blocking the interaction between c-Ski and DNA binding domain [21]. Two point mutations in the TA domain localized in close proximity to c-Ski binding site could also have detrimental effect on c-Ski binding. CBP/p300 was also shown to act as bridge between c-Myb and C/EBPβ [23]. Therefore, we can speculate that it may play the same role for c-Myb and Mybbp 1a.

In our study, we also showed that c-Myb is downregulated in myogenic cells by its 3′ untranslated region. We presumed that miR-150 could be responsible for downregulation, but its expression was low, only slightly upregulated and soon lowered during differentiation. As our data indicate that proper downregulating of c-Myb is vitally important for accomplishing myogenic differentiation, we speculated that several miRNAs could cooperate in inhibiting c-Myb expression. We therefore searched in the literature if some of miRNAs that have already been described to downregulate c-Myb via interacting with 3′ UTR, (miR-150, miR-15a, miR-34a, miR-126, miR-200b, miR-200c and miR-429), were activated during muscle differentiation. In [24] there is described that some quoted miRNAs were continuously upregulated during differentiation of C2C12 cells from D24 to D72 compared to GM values: miR-15a (from 0.92 times to 2.7 times), miR-126 (from 1.95 times to 2.75 times) and miR-200b (from 1.75 times to 2.73 times), these miRNAs could therefore play a role in extinguishing c-Myb expression. To our surprise, miR-15a and miR-126, two candidates miRNAs for targeting c-Myb 3′ UTR in differentiating cells, are also downregulated in a more metastatic alveolar form (ARMS) of rhabdomyosarcoma, tumors with myogenic features [25]. Moreover, in some ARMS, an elevated level of c-Myb transcript was detected [26]. A more detailed analysis of the c-Myb protein level in ARMS is needed, but our data indicate that c-Myb has the ability to contribute to the tumor phenotype in ARMS.

To summarize our results, we suggest a new role for c-Myb as a regulator of myogenic differentiation.

## Supporting Information

Figure S1 **Representative confocal image of C2C12 cells infected with c-Myb-RET.** Cells were immunostained for c-Myb and eGFP showing the co-expression of both genes in the same cell. DAPI staining identifies nuclei.
(TIF)

Figure S2 **Cultivation of isolated myofibers.** Isolated myofibers (F) were cultivated in enriched growth medium on Matrigel and satellite cells emigrating from myofiber were immunostained for MyoD after 5 or 7 days in culture. DAPI staining identifies nuclei. We note that proliferating myoblasts (MyoD positive) are viable and formed multinucleated myotubes after 7 days in culture even though the original myofiber collapsed. A representative image is shown.
(TIF)

Figure S3 **Regeneration of cardiotoxin-injured tibialis anterior muscle.** 7 µm cross-sections of cardiotoxin-injected TA muscle taken from untreated, 5 and 7 days postcardiotoxin-injured muscle were stained with hematoxylin and eosin.
(TIF)

Figure S4 **Expression of miR-150 in C2C12 cells.** Relative expression levels of miR-150 in growing C2C12 cells (GM) differentiating for 24 hours (D24) and 72 hours (D72) normalized to U6 snRNA.
(TIF)

Table S1 **Primers used for PCR.**
(DOC)

Movie S1 **Time lapse imaging of cultured myofibers.** Myofibers were cultured in growth medium on Matrigel and imaged every 3 minutes for 5 days starting at day two of myofiber cultures.
(AVI)

Movie S2 **Time lapse imaging of cultured myofibers infected with c-Myb-RET.** Myofibers cultured in enriched growth medium on Matrigel were infected with c-Myb expressing retrovirus. Cells co-expressing retrovirus were monitored, as eGFP-positive cells visible in fluorescence images that were taken together with phase contrast images every 3 minutes for 3 days starting at day four of myofiber cultures. The entire time-lapse movie is provided as the merged images of phase contrast and eGFP fluorescence images.
(AVI)

Movie S3 **Time lapse imaging of cultured myofibers infected with control-RET.** Myofibers were infected with control empty retrovirus and analyzed as described in Movie S2.
(AVI)
