# Supplementary figures and images for: c-Myb Inhibits Myoblast Fusion
Source: PLoS One. 2013 Oct 21;8(10):e76742. doi: 10.1371/journal.pone.0076742 (PMC3804598; doi:10.1371/journal.pone.0076742)

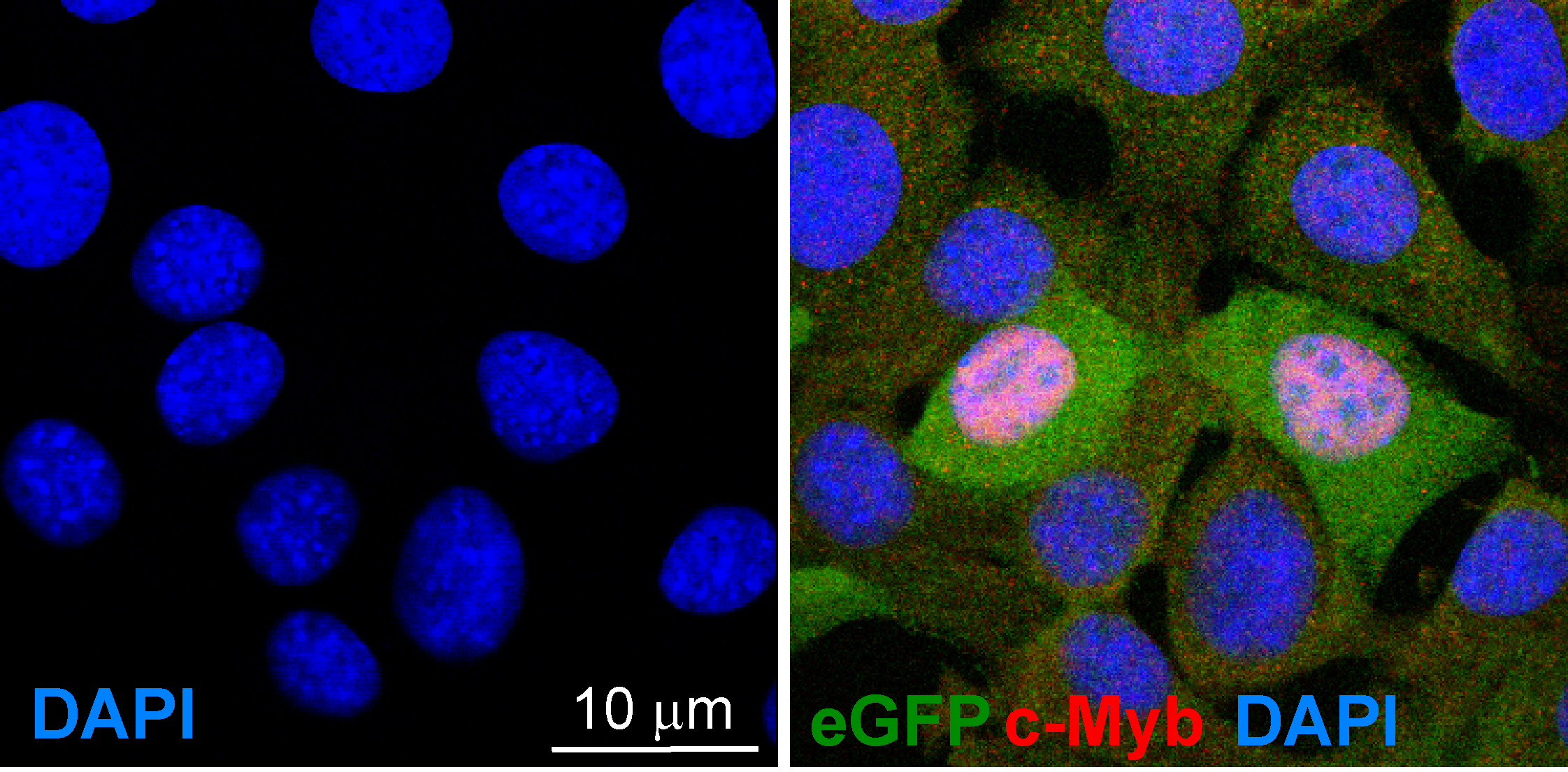

Supplement: Figure S1 — Representative confocal image of C2C12 cells infected with c-Myb-RET. Cells were immunostained for c-Myb and eGFP showing the co-expression of both genes in the same cell. DAPI staining identifies nuclei. (TIF) [file pone.0076742.s001.tif]

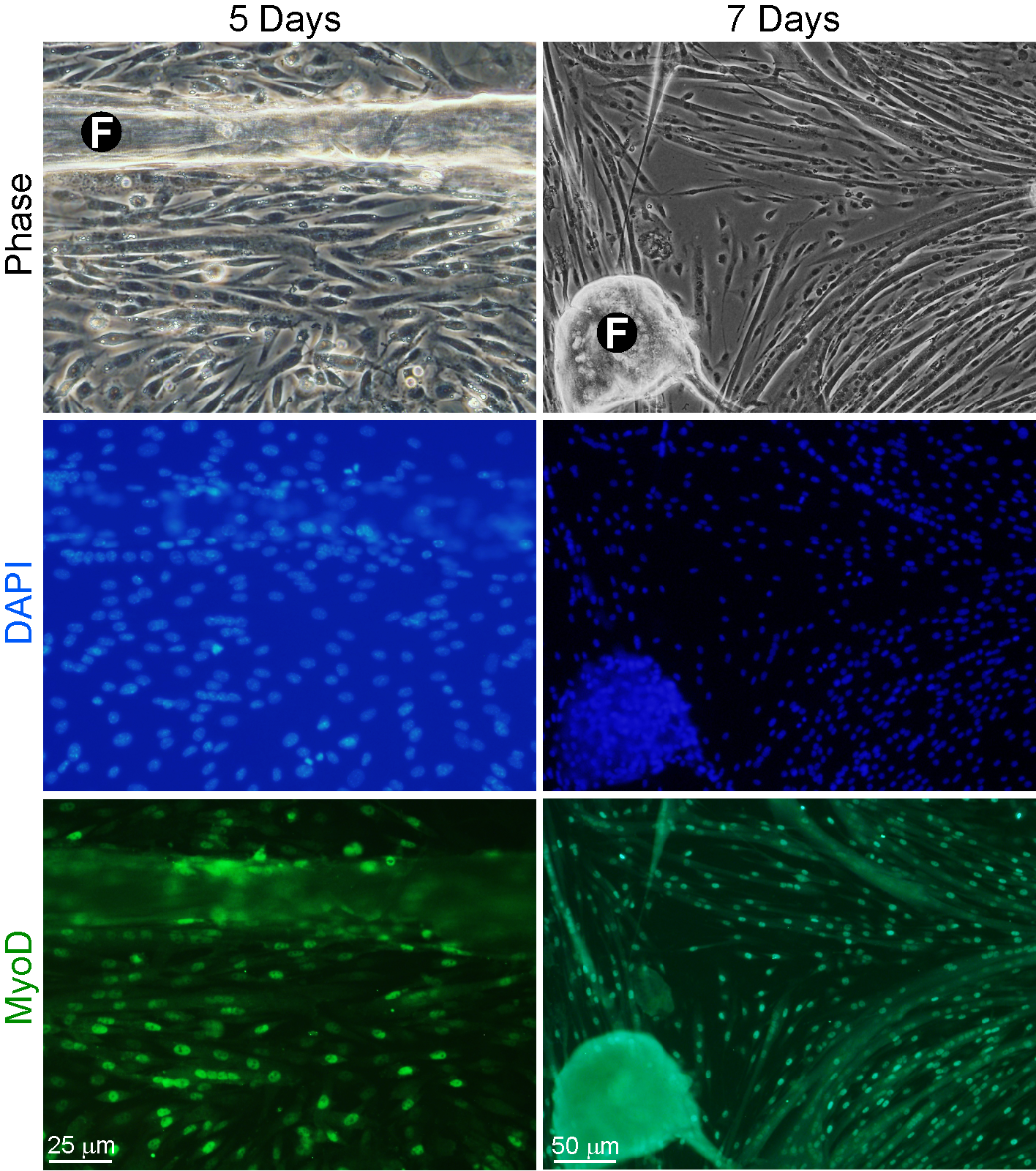

Supplement: Figure S2 — Cultivation of isolated myofibers. Isolated myofibers (F) were cultivated in enriched growth medium on Matrigel and satellite cells emigrating from myofiber were immunostained for MyoD after 5 or 7 days in culture. DAPI staining identifies nuclei. We note that proliferating myoblasts (MyoD positive) are viable and formed multinucleated myotubes after 7 days in culture even though the original myofiber collapsed. A representative image is shown. (TIF) [file pone.0076742.s002.tif]

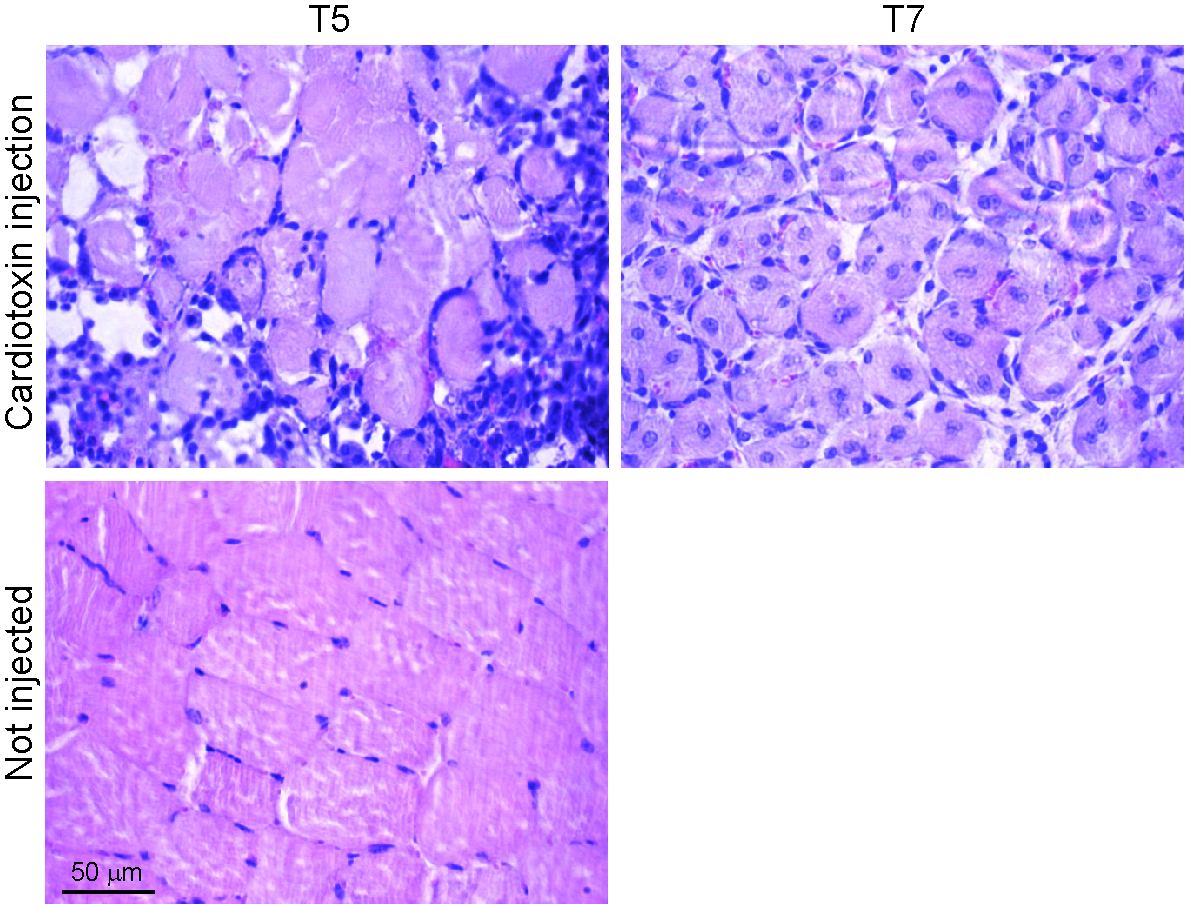

Supplement: Figure S3 — Regeneration of cardiotoxin-injured tibialis anterior muscle. 7 µm cross-sections of cardiotoxin-injected TA muscle taken from untreated, 5 and 7 days postcardiotoxin-injured muscle were stained with hematoxylin and eosin. (TIF) [file pone.0076742.s003.tif]

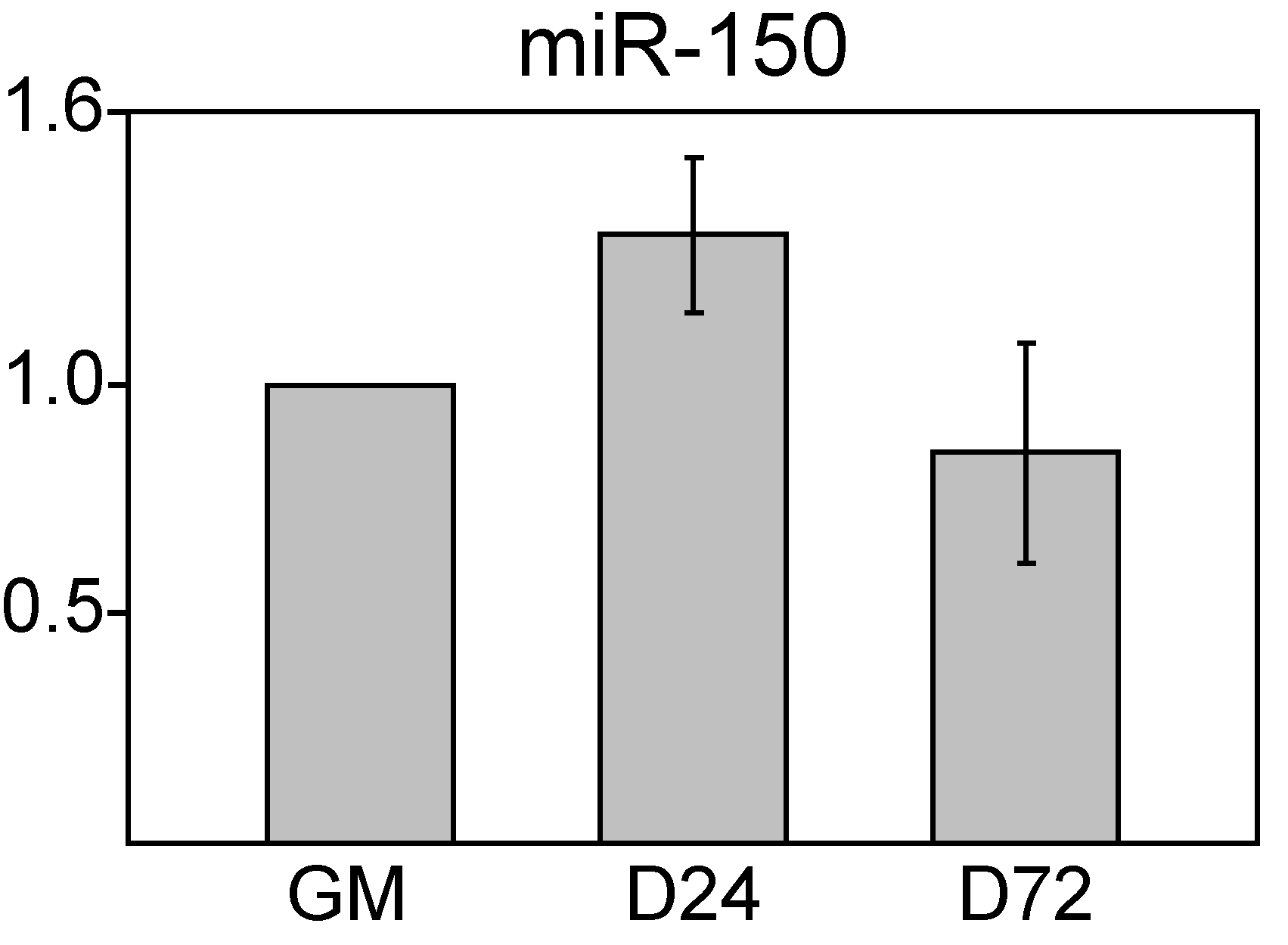

Supplement: Figure S4 — Expression of miR-150 in C2C12 cells. Relative expression levels of miR-150 in growing C2C12 cells (GM) differentiating for 24 hours (D24) and 72 hours (D72) normalized to U6 snRNA. (TIF) [file pone.0076742.s004.tif]
