# Supplementary material for: c-Myb Inhibits Myoblast Fusion
Source: PLoS One. 2013 Oct 21;8(10):e76742. doi: 10.1371/journal.pone.0076742 (PMC3804598; doi:10.1371/journal.pone.0076742)
Supplement: Table S1 — Primers used for PCR. (DOC) [file pone.0076742.s005.doc]

| **Table S1. qRT-PCR primers and RT-PCR primers** | | | |
| --- | --- | --- | --- |
| **Gene** |  | **Sequence 5´ > 3´** | **Amplicon size (nt)** |
| LRRN1 | Sense | cccagccttaggacacgtctc | 220 |
|  | Antisense | cgattcagtggcaaggagaacag |  |
| MYOG | Sense | ggtcccaacccaggagatca | 185 |
|  | Antisense | gacaccaacttgggggctca |  |
| TNNC2 | Sense | ccgcatctttgacaggaacgca | 188 |
|  | Antisense | ctccttactgaacgccctccat |  |
| GAPDH | Sense | AACTTTGGCATTGTGGAAGG | 69 |
|  | Antisense | ATCCACAGTCTTCTGGGTGG |  |
